# The critical-thinking paradox in generative AI-integrated learning: distinguishing efficiency from cognitive depth—a differentiated framework and testable propositions

**DOI:** 10.3389/fpsyg.2026.1906070

**Published:** 2026-08-14

**Authors:** Jiayin Lin, Naif Mohammed Al-Hada

**Affiliations:** 1 Guangxi Normal University, Guilin, China; 2 Baise University, Baise, China

**Keywords:** AI literacy, cognitive debt, cognitive load theory, cognitive offloading, critical thinking, deep and surface learning, desirable difficulties, differentiated instruction

## Abstract

Generative artificial intelligence (GenAI) has rapidly entered educational settings, yet a fundamental question remains unresolved: does GenAI enhance learning or does it improve immediate performance while reducing the cognitive activity on which durable learning depends? This Hypothesis and Theory article offers a theoretical reading of apparently contradictory findings—improved academic products alongside qualitative, neuroscientific, and behavioral signs of reduced cognitive engagement—which we term the critical-thinking paradox of GenAI-integrated learning. These contrasts may also reflect genuine heterogeneity across tasks, populations, and tools; the proposed convergence is treated as a testable interpretation, not a fact. Drawing on four theoretical traditions—levels of processing, desirable difficulties, cognitive load theory and Load Reduction Instruction, and cognitive offloading research—we propose a differentiated three-level framework that maps AI-integration strategies onto surface, intermediate, and deep cognitive processing, specifying level-appropriate AI roles, primary risks, and boundary conditions. We adopt the emerging construct of cognitive debt: a potential cumulative reduction in metacognitive calibration and unaided higher-order performance that persists beyond an AI-assisted episode. Our contribution is to distinguish episodic offloading (deliberate and task-specific) from habitual offloading (routine and weakly monitored) and to map both patterns onto the three cognitive levels. The framework generates falsifiable hypotheses, centrally that unrestricted AI use on deep-processing tasks may yield a product–process dissociation: higher-rated assignments but lower unaided delayed transfer. We specify developmental stage, prior knowledge, and metacognitive monitoring accuracy as preregistered boundary conditions and outline a research program combining confirmatory experiments, interaction telemetry, and longitudinal measurement.

## 1 Introduction

Generative artificial intelligence (GenAI) has entered educational settings at a pace that has substantially outpaced the development of evidence-based pedagogical frameworks for its integration. Within approximately 2 years of the public release of large language models (LLMs), such as ChatGPT, these tools have transitioned from experimental curiosities to routinely used learning aids across all educational levels. Systematic reviews indicate that adoption has been rapid, widespread, and predominantly *ad hoc*, with insufficient attention to the management of cognitive load and the systematic differences between novice and expert learners (Kasneci et al., 2023; Kundu and Bej, 2025; Martin et al., 2025). Educational psychology—the discipline charged with generating and applying knowledge about how people learn—now confronts an urgent theoretical and empirical question: is GenAI enhancing learning, or is it enhancing performance while quietly eroding the cognitive processes upon which genuine, durable, transferable learning depends?

The first wave of empirical studies, concentrated in 2024–2025, presents a mixed picture whose interpretation remains open. Variation across tasks, populations, GenAI systems, instructional supports, and outcome measures may represent genuine heterogeneity rather than a single underlying effect. We nevertheless examine whether part of this variation can be interpreted through a common product–process distinction. On one hand, quasi-experimental evidence indicates that AI-assisted students can achieve higher academic scores than peers using traditional resources (Mariñoso et al., 2025), and survey research suggests that positive attitudes toward GenAI mediate the relationship between teacher support and learner well-being (Pan and Li, 2025). A meta-analysis of 56 experimental studies reports a moderate positive short-term effect of AI-supported learning on creative thinking (standardized mean difference = 0.54; Peng et al., 2025). On the other hand, process-oriented mixed-methods research identifies limitations in critical-thinking depth and compensatory verification behaviors in response to weaknesses in AI-generated content (Wang and Wu, 2025). Teacher-education studies similarly report moderate AI awareness alongside weaker problem-solving, critical-evaluation, and pedagogical-integration competencies (Han, 2025; Kazmaci et al., 2025).

A recent contribution by Jose et al. (2025) provided an important conceptual advance by framing this dual effect as a cognitive paradox between enhancement and erosion, grounding the analysis in cognitive load theory, Bloom’s taxonomy, and self-determination theory. While we concur with their diagnosis and their call for balanced AI integration, we contend that the field requires a more granular, operationalized framework—one that moves beyond the binary question of whether GenAI is helpful or harmful and instead specifies the conditions under which AI integration serves versus undermines specific learning objectives at specific cognitive processing levels.

The present article advances this discourse through five contributions. First, we synthesize the 2024–2025 evidence base—spanning product-outcome studies, process-sensitive qualitative research, teacher-competency investigations, and emerging neuroscientific findings—to develop a theoretically interpretable account of the mixed findings, while retaining genuine heterogeneity as a rival explanation. Second, we integrate four established theoretical traditions—the levels-of-processing framework (Craik and Lockhart, 1972), desirable difficulties (Bjork and Bjork, 2011), cognitive load theory (Sweller et al., 2011) as extended by Load Reduction Instruction (Martin et al., 2025), and cognitive offloading research (Risko and Gilbert, 2016; Skulmowski, 2023)—to explain when product gains may diverge from durable learning. Third, we propose a differentiated three-level framework that maps AI-integration strategies onto surface, intermediate, and deep cognitive processing, with *a priori* classification rules, level-specific AI roles, risks, and boundary conditions. Fourth, we adopt the emerging cognitive-debt construct, attribute its prior use, and extend it through an episodic–habitual offloading distinction and a three-level mapping. Fifth, we derive falsifiable hypotheses, outline a confirmatory experimental design, and specify concrete operational telemetry—including chat-log prompt dynamics and Experience Sampling Methodology—to track real-time shifts in offloading. The article concludes with implications for teacher education and educational policy.

The structure of the article is as follows: Section 2 reviews four domains of evidence and develops the critical-thinking paradox as one testable interpretation of a heterogeneous literature; Section 3 presents the theoretical foundations; Section 4 elaborates the differentiated three-level framework—it is *a priori* task-classification rule, and boundary conditions; Section 5 formalizes the propositions into testable hypotheses and develops the episodic–habitual offloading distinction and cognitive-debt construct; Section 6 specifies methodological implications, including real-time operational telemetry; Section 7 discusses implications for teacher education and educational policy; finally, Section 8 offers a brief conclusion.

## 2 The critical thinking paradox: a review of converging evidence

In this section, we review the emerging evidence base on GenAI in education across four domains. We ask whether selected findings can be interpreted as a product–process dissociation—the critical-thinking paradox—while recognizing that variation in tasks, populations, tools, and designs may also produce genuine heterogeneity. The synthesis is therefore a theory-guided reading to be tested, not a claim that the literature has already established one uniform pattern.

### 2.1 The efficiency evidence: performance gains are real but potentially superficial

The most straightforward evidence for GenAI’s educational benefits emerges from studies employing product-oriented outcome measures. Mariñoso et al. (2025) in a sequential explanatory mixed-methods investigation with 238 undergraduate early-childhood-education students, found that an AI-assisted group achieved significantly higher academic scores designing mathematical learning situations compared with a control group using traditional resources. From a cognitive-load perspective, such gains are theoretically plausible: GenAI can reduce extraneous processing demands—the mental effort directed at navigating tasks rather than learning from them—theoretically freeing learners to allocate cognitive resources to germane processing and higher-level conceptual work (Sweller et al., 2011). Similarly, Pan and Li (2025) demonstrated that positive attitudes toward GenAI mediated the relationship between teacher support and learner well-being among 486 Chinese university EFL learners, a finding consistent with the expectation that AI tools supporting learners’ needs for autonomy, competence, and relatedness can enhance intrinsic motivation (Ryan and Deci, 2000; Chiu, 2024). Peng et al. (2025) in a meta-analysis of 56 experimental studies, reported a moderate positive short-term effect of AI-supported learning on creative thinking (SMD = 0.54). However, the authors noted substantial heterogeneity in effect sizes and a severe under-representation of long-term follow-up assessments.

However, these studies share a critical methodological feature that bounds the interpretability of their affirmative conclusions: their primary outcome measures capture product quality—assignment grades, self-reported attitudes, and perceived well-being. They answer the question ‘Did AI help students produce better work?’ with a qualified yes, but they do not answer the complementary question “Did AI help students learn in a manner that produces durable, transferable knowledge?” Martin et al. (2025) have recently cautioned that GenAI adoption has proceeded *ad hoc*, with insufficient attention to managing cognitive load or accounting for individual differences between novice and expert learners. The concern is amplified by the well-established finding in cognitive psychology that the conditions enhancing performance during acquisition frequently diverge from—and may oppose—those enhancing long-term retention and transfer (Soderstrom and Bjork, 2015).

### 2.2 The shallow-processing concern: evidence from process-sensitive and multimodal research

When researchers shift their methodological lens from product to process, a more troubling picture emerges. Wang and Wu (2025), employing a mixed-methods design with 363 undergraduate design students interacting with a domain-specific AI teaching assistant, identified three qualitative themes that we argue are paradigmatic of the critical thinking paradox: (a) students valued AI’s accessibility and efficiency; (b) students identified gaps in depth, interpretation, and critical thinking; and (c) students described engaging in critical trust and verification behaviors—cross-checking AI outputs against external sources because they sensed the tool’s interpretive shallowness. Critically, students deployed metacognitive strategies not to deepen their own cognitive processing but to compensate for the perceived limitations of the AI. This pattern converges with the long-standing distinction between deep and surface approaches to learning (Marton and Säljö, 1976; Entwistle and Ramsden, 1983): the efficiency of AI may inadvertently promote surface-level processing by supplying ready-made answers that allow learners to bypass the cognitive struggle on which deep conceptual understanding depends.

Choudhuri et al. (2026) reported a preprint analysis of survey data from 299 STEM students across five North American universities. Using Partial Least Squares Structural Equation Modeling, they found that trust-driven routine GenAI use was negatively associated with reflection, critical thinking, and need for understanding. Students with higher technophilic motivations and greater computer self-efficacy showed stronger associations with the reported disengagement pattern. Because the design is observational and cross-sectional, the paths do not establish that routine AI use causes declining engagement or that a self-reinforcing cycle develops over time. The authors proposed that the cognitive-debt cycle is therefore treated here as a longitudinal mechanism requiring direct prospective testing rather than as an observed causal sequence.

Neuroscientific evidence is suggestive but preliminary. In a preprint electroencephalography study, Kosmyna et al. (2025) reported weaker functional connectivity during AI-assisted essay writing than during search-engine-assisted or unaided writing in an initial sample of 54 participants; 18 participants completed the crossover session. After the AI tool was removed, the LLM-first subgroup retained some indicators of lower engagement. These results are consistent with a persistence effect but do not establish dependency or cumulative cognitive impairment. Complementary peer-reviewed behavioral evidence comes from Barcaui’s (2025) randomized controlled trial: 120 undergraduates studied unfamiliar material with unrestricted ChatGPT or traditional methods and completed a surprise retention test 45 days later. The ChatGPT condition scored lower on retention (57.5% vs. 68.5%; t(83) = −3.19, *p* = 0.002, Cohen’s d = 0.68) despite shorter study time. This single-institution business-student trial is the closest current behavioral analog to the proposed dissociation, not a complete test of it, because product quality and unaided deep transfer were not jointly assessed within the same learners.

An independent peer-reviewed field experiment broadens the population and domain. Bastani et al. (2025) studied nearly 1,000 high-school students in mathematics and found that access to a general-purpose GPT interface improved performance during supported practice but was followed by poorer unaided test performance after access was removed; a guarded tutor designed to preserve learning mitigated that loss. The findings strengthen the case for distinguishing immediate assistance from unaided learning and for treating AI design as a moderator. They do not by themselves establish the proposed three-level mechanism.

Additional evidence comes from the teacher-education domain. Han (2025), assessing 79 pre-service teachers across six AI-competency domains following structured AI coursework, found that participants scored highest in AI awareness and ethics but lowest in problem-solving, basic AI skills, and AI teaching practice. This competency gap—knowing about AI without knowing how to think with AI—mirrors the student-level paradox: surface familiarity with AI tools masks deeper cognitive and pedagogical disengagement.

The developmental trajectory of cognitive offloading further nuances the shallow-processing concern; because this dimension is central to the framework, we treat it separately in Section 2.4.

At the tertiary level, Yang et al. (2024) provided an important qualitative framework by analyzing interviews, GenAI chat logs, and reflective journals from 74 postgraduate students across three Chinese universities. The study identified four types of student agency—receptive, resistive, resourceful, and reflective—and three overarching perspectives: beneficial optimism, cautious skepticism, and adaptable pragmatism. The authors positioned student agency as the mechanism through which surface or deep learning approaches emerge in GenAI-mediated environments. While we build upon this agency-centered premise, our framework shifts the unit of analysis from the learner’s disposition (agency type) to the task’s cognitive demand (processing level), rendering the framework directly operationalizable for instructional design.

### 2.3 The mediation gap: teacher and learner psychology as bottlenecks

A third body of evidence concerns the mechanisms through which AI knowledge translates—or fails to translate—into effective educational practice. Kazmaci et al. (2025) employed structural equation modeling with 340 primary school teachers in Northern Cyprus to demonstrate that theoretical and practical AI knowledge contributed to sustainable AI-integration ability only indirectly, with teachers’ beliefs and attitudes serving as critical mediators. Knowledge alone was insufficient; what determined integration was whether teachers believed AI to be valuable and felt confident deploying it. This mediation finding converges with a growing body of research on AI-TPACK—the extension of technological pedagogical content knowledge (Shulman, 1986; Mishra and Koehler, 2006) into the GenAI era. Wang et al. (2024), surveying 312 K-12 in-service teachers in China, demonstrated that AI literacy was not statistically associated with teachers’ behavioral intentions to design AI-assisted learning directly; rather, TPACK significantly mediated the path from AI literacy to behavioral intentions. Al-Abdullatif (2024) reported parallel findings among 237 university teachers: AI literacy and perceived ease of use were the most influential factors for GenAI acceptance, with Intelligent TPACK and perceived trust functioning as pivotal mediators. Chen et al. (2025), employing structural equation modeling with 1,015 Chinese middle-school teachers, provided further support by demonstrating that professional capital influenced ICT integration only through the sequential mediation of constructivist belief, differentiated instruction, and AI literacy. The consistent pattern across these studies is that the bottleneck is not technical competence—as much professional-development discourse assumes—but psychological readiness: self-efficacy, pedagogical beliefs, and perceived compatibility between AI tools and educational values.

At the student level, Montag and Elhai (2025) provided preliminary evidence from two independent samples—a German general-population sample (*n* = 266) and a US student sample (*n* = 283)—that deep learning style mediates the relationship between AI competence and adaptive personal enhancement tendencies. This mediation was significant in the German sample, where individuals scoring higher on deep learning style (as measured by the short version of the Study Process Questionnaire) showed stronger positive associations between AI competence and adaptive AI use. In the US student sample, the full mediation model did not replicate; a significant indirect effect emerged only in the male subsample, with small-to-medium effect sizes. These cross-cultural and gender-specific differences underscore the need for theoretical frameworks that can accommodate individual-difference moderators. If students form positive AI attitudes chiefly through uncritical exposure and efficiency experiences, they may be ill-equipped to recognize when AI use becomes cognitively counterproductive. The field has not yet developed the conceptual tools to distinguish productive AI attitudes—those enabling strategic, metacognitive, context-sensitive use—from unproductive ones that equate AI fluency with learning.

The cognitive architecture of digital externalization provides a mechanistic account of this gap. Skulmowski (2023) proposed a model in which cognitive load functions as the central trigger for offloading behavior: high intrinsic load, high extraneous load, and low perceived working memory capacity all increase the probability that learners will externalize cognitive operations to digital tools. The model further warns that offloading can create an illusion of having memorized information when only the gist or retrieval location has been retained—a metacognitive blind spot that is particularly dangerous in AI-mediated learning, where the fluency and surface plausibility of AI-generated content may amplify the illusion of understanding.

### 2.4 The developmental dimension: age-related differences in cognitive offloading

A dimension largely absent from existing AI-integration frameworks is developmental stage. Sun et al. (2025), using a word-pair memory task, documented a developmental shift in cognitive offloading: primary school children (approximately ages 7–11) tended to under-use external cognitive aids, relying on internal memory even when offloading would have improved performance, whereas secondary school students (approximately ages 12–15) tended to over-rely on external aids at the expense of internal effort. Metacognitive-monitoring accuracy was associated with offloading bias in the younger group but not the older group, suggesting that different mechanisms govern offloading at different developmental stages.

Armitage and Gilbert (2025), in a review of the development of cognitive offloading in children, argued that the capacity for selective, strategic offloading—knowing when to externalize and when to rely on internal processing—develops gradually through middle childhood and adolescence and is tied to the maturation of metacognitive and executive function systems. Convergent evidence that technology effects are never uniform comes from Jing et al. (2023), whose meta-analysis in child development showed that the influence of screen media on young children’s vocabulary depends heavily on content quality, interactivity, and adult scaffolding. Together, these findings imply that the framework’s risk gradient should be developmentally calibrated rather than applied uniformly across grade levels.

### 2.5 Bibliometric context: the convergence of theoretical and methodological trajectories

To contextualize these theoretical and methodological trajectories, we conducted a revision-stage verification of a Scopus scoping review using 12 finalized queries on 25 July 2026. The queries yielded 10,246 raw records; DOI-first and normalized-title deduplication produced 8,869 unique records, and prespecified document-type, English-language, Social Sciences Citation Index (SSCI)-whitelist, and title/abstract screening retained 969 publications. Keyword-coded distributions indicated that metacognition and self-regulated learning, critical thinking, cognitive load, cognitive offloading, and self-determination were prominent and rapidly expanding topics, while structural equation modeling, mixed methods, and controlled intervention designs were increasingly represented. We take these patterns as circumstantial support for the timeliness of the present synthesis rather than as direct evidence for any psychological claim. The full finalized search strategy, exact screening pipeline, coding schemes, and VALOR-standard reproducibility audit are provided in the Supplementary material; the verified dataset is available from the authors on reasonable request.

### 2.6 Synthesis: four converging lines of evidence

Taken together, these four domains can be read as a theoretically coherent pattern, but genuine heterogeneity remains a viable rival explanation. Differences in task demands, learner age and expertise, model affordances, scaffolding, and outcome measures may generate different effects without a single paradoxical mechanism. We therefore treat the critical-thinking paradox as a falsifiable interpretation: under specified conditions, GenAI may improve academic product quality while reducing cognitive engagement, metacognitive calibration, or unaided delayed transfer. The efficiency and cognitive-cost findings are not assumed to be two sides of the same coin in every setting; the framework predicts when they should co-occur and can be challenged by within-study evidence that product gains are accompanied by intact or improved transfer. The following sections develop that theoretical account and the confirmatory designs needed to distinguish it from heterogeneity-based alternatives.

## 3 Theoretical foundations

The differentiated framework we propose is grounded in four established theoretical traditions, each of which captures a distinct mechanism through which GenAI may produce divergent effects on performance and learning. We briefly review each tradition and specify its contribution to the framework.

### 3.1 Levels of processing and the deep–surface distinction

The levels-of-processing (LoP) framework (Craik and Lockhart, 1972) established that the durability of memory traces depends on the depth of cognitive processing at encoding: shallow processing (e.g., perceptual or phonetic analysis) produces fragile, rapidly decaying traces, whereas deep processing (e.g., semantic elaboration and meaningful integration) produces robust, long-lasting traces. This distinction was extended to educational contexts through the concepts of deep and surface approaches to learning (Marton and Säljö, 1976; Entwistle and Ramsden, 1983; Biggs et al., 2001). A surface approach involves reproducing content with minimal elaboration—memorization, rote rehearsal, and passive acceptance of information—while a deep approach involves seeking meaning, relating new ideas to prior knowledge, and critically examining evidence. GenAI presents a novel threat to deep processing because it supplies semantically elaborated content without requiring the learner to perform the elaborative cognitive operations. The student receives the product of analysis without engaging in the process of analysis—a pattern that, in levels-of-processing terms, constitutes shallow encoding despite the surface sophistication of the output.

Although the levels-of-processing framework is rarely invoked by name in the current AI-education literature—a scarcity that partly reflects terminology, since the phrase “deep learning” now denotes a machine-learning method rather than a depth-of-processing construct—it provides a uniquely powerful lens for diagnosing the critical thinking paradox: GenAI supplies semantically elaborated output without requiring semantic elaboration by the learner. This makes LoP not merely a historical foundation but a diagnostically precise tool for distinguishing between genuine deep processing and its AI-generated simulation.

### 3.2 Desirable difficulties

The desirable difficulties framework (Bjork, 1994; Bjork and Bjork, 2011) holds that introducing challenges during learning—such as spaced practice, interleaving, generation effects, and retrieval practice—may lower immediate performance but enhance long-term retention and transfer. Conversely, conditions that make learning feel easy and fluent often produce illusions of competence, wherein learners overestimate their mastery. GenAI, by providing fluent, well-structured, immediately accessible answers, systematically removes the desirable difficulties that consolidate long-term memory and promote deep understanding. When a student asks ChatGPT to summarize a chapter, explain a concept, or propose a solution, the AI performs the generation, elaboration, and synthesis operations that the student should be practicing. The fluency of the AI output may produce high confidence (Pan and Li, 2025), but this confidence may be illusory if it is not accompanied by the internal cognitive operations that produce durable learning (Koriat and Bjork, 2005).

The desirable difficulties framework is similarly seldom invoked in the current AI-education literature. Our framework reintroduces it as an essential corrective—a principled reminder that the conditions maximizing immediate performance often differ from those maximizing durable understanding (Soderstrom and Bjork, 2015).

### 3.3 Cognitive load theory and load reduction instruction

Cognitive load theory (Sweller et al., 2011, 2019) distinguishes among intrinsic cognitive load (inherent to the learning material), extraneous cognitive load (imposed by suboptimal instructional design), and germane cognitive load (productive effort directed at schema construction and automation). Martin et al. (2025) recently extended cognitive load theory into the GenAI context through the Load Reduction Instruction (LRI) framework, which proposes that GenAI can reduce extraneous load—for example, by handling information retrieval, formatting, and basic summarization—thereby freeing working memory resources for germane processing. The critical implication, which we develop as a core proposition of our framework, is that the pedagogical value of GenAI depends on whether the cognitive operations it offloads are extraneous (their removal benefits learning) or germane (their removal impairs learning). When GenAI offloads germane processing—the cognitive work of organizing, elaborating, evaluating, and synthesizing—it may improve immediate performance while undermining the very learning it is intended to facilitate. This generates the novel prediction, elaborated in Section 5, that the educational effects of GenAI are fundamentally task-level contingent.

Self-determination theory (Ryan and Deci, 2000) adds a crucial motivational dimension to the CLT-LRI account. When learners exercise autonomous choice over when and how to deploy AI, the cognitive-load-reducing effects of AI are experienced as competence-supportive rather than as externally imposed simplification. Conversely, when AI use is mandated or becomes habitual, the same load reduction may undermine the basic psychological needs for autonomy and competence, paradoxically reducing intrinsic motivation for the very learning that AI was intended to support. The integration of self-determination theory (SDT) with cognitive load theory (CLT) thus yields a more complete prediction: the pedagogical value of AI-assisted load reduction is moderated by the degree to which the learner experiences AI use as autonomous and competence-congruent. This insight is directly operationalized in our episodic-habitual offloading distinction (Section 5.2), where the transition from strategic to automatic AI use corresponds to a shift from autonomy-supportive to autonomy-undermining cognitive externalization.

### 3.4 Cognitive offloading: from adaptive strategy to cognitive debt

Cognitive offloading refers to the use of external tools to reduce internal cognitive demands (Risko and Gilbert, 2016). Offloading is a behavior, not an impairment, and it is often adaptive. Experimental literature nevertheless identifies conditions under which it may carry costs: when it reduces encoding effort (Grinschgl et al., 2021), creates an illusion of knowledge (Fisher et al., 2015), or becomes routine and weakly monitored (Choudhuri et al., 2026). Skulmowski (2023) proposed that high intrinsic or extraneous load and low perceived or actual working-memory capacity can trigger digital externalization, with a risk that learners retain only the gist of externally stored information while believing that they have internalized it.

GenAI may extend the scope of cognitive offloading because, unlike calculators, search engines, or notebooks, it can perform synthesis, reasoning, evaluation, and creative ideation—the operations that often constitute higher-order learning objectives. Following Kosmyna et al. (2025), we use cognitive debt to denote a potential cumulative reduction in metacognitive calibration and/or unaided higher-order cognitive performance that persists beyond a particular AI-assisted episode. We do not claim to coin the term, and we treat the cycle proposed by Choudhuri et al. (2026) as a hypothesis. Our original extension is the distinction between episodic offloading—the deliberate, task-specific delegation of an operation while retaining awareness of what is offloaded and why—and habitual offloading—routine, weakly monitored reliance that may recalibrate expected cognitive effort—and the mapping of these patterns onto surface, intermediate, and deep tasks.

For construct validity, cognitive debt must be separated from its antecedents and possible mechanisms. Offloading frequency and prompt behavior are exposure measures; disuse-related weakening is a hypothesized mechanism; and dependency describes a pattern of reliance or difficulty functioning without the tool. Cognitive debt, by contrast, should be operationalized as a within-person deterioration from baseline in outcomes measured without AI—especially delayed transfer and item-level monitoring calibration—after controlling for prior knowledge and practice. A study should therefore be able to observe frequent offloading without debt, or debt without high concurrent use; otherwise, the construct would be circular.

Underpinning all four theoretical traditions is a common mechanism: metacognitive monitoring. Whether a learner engages in deep or shallow processing depends on accurate monitoring of their own comprehension. Whether a difficulty is experienced as desirable or merely frustrating depends on the learner’s capacity to detect the gap between current and target understanding. Whether cognitive load is extraneous or germane can only be evaluated through metacognitive calibration of task demands against available resources. And whether offloading remains episodic or becomes habitual depends on the learner’s awareness of what has been offloaded and why. Metacognition is therefore not merely one theoretical tradition among others—it is the cross-cutting mechanism through which the other four frameworks exert their effects on learning. We develop this proposition in Section 4.5.

These four theoretical traditions are not competing accounts but complementary lenses on a common underlying mechanism. Levels of processing specifies what is lost when AI supplies semantically elaborated content without learner elaboration; desirable difficulties specifies why making learning easier undermines durable retention; cognitive load theory specifies the operational distinction between productive and counterproductive offloading; and cognitive offloading research specifies the transition from strategic tool use to habitual dependency. Together, they provide the foundation for the differentiated framework to which we now turn.

## 4 The differentiated framework: a three-level model of AI integration

**Figure 1:**
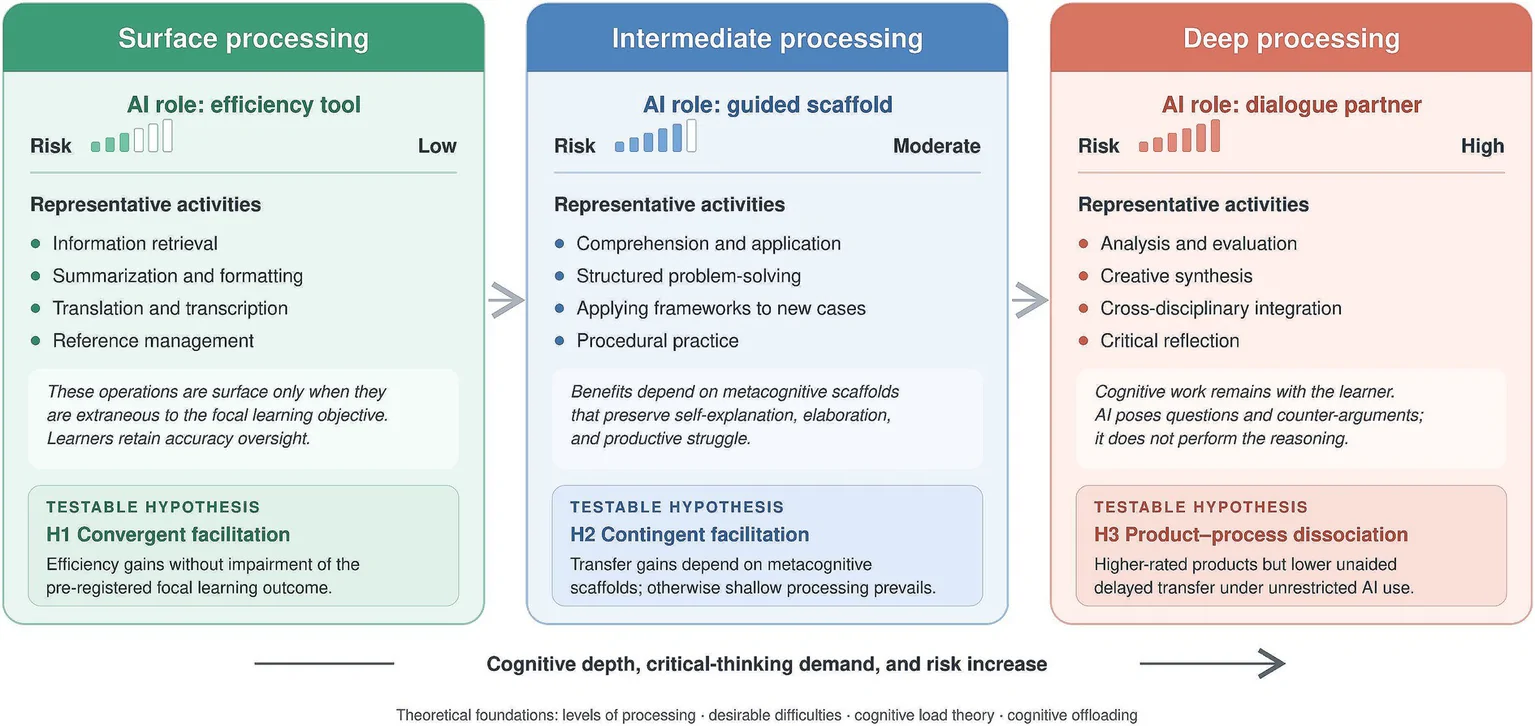
A differentiated framework for generative AI integration by cognitive-processing level. For each level (surface, intermediate, and deep), the panel specifies the appropriate AI role, risk of uncritical use, representative activities, and testable hypothesis. Classification is relative to the preregistered focal learning objective rather than to an activity label alone. Cognitive depth, critical-thinking demand, and risk increase from left to right.

Under the theoretical interpretation developed above, the effects of GenAI should depend on the relation between the operation delegated to AI and the focal learning objective. We propose a differentiated three-level framework (Table 1; Figure 1) that maps AI-integration strategies onto surface, intermediate, and deep cognitive processing. For each level, the framework specifies (a) the cognitive operations involved, (b) the appropriate role for AI, (c) the primary risks of uncritical use, (d) testable predictions, and (e) preregistered boundary conditions.

**Table 1:** A differentiated framework for GenAI integration by cognitive-processing level.

Cognitive level	Representative activities	AI role and strategy	Primary risk	Testable hypothesis
Surface (retrieval, summarization, formatting, translation, and transcription)	Literature searches, note organization, language polishing, reference formatting, and translation	AI as efficiency tool: reduce extraneous load to free working memory for higher-order processing	Low when the delegated operation is extraneous to the focal learning objective; accuracy oversight remains necessary	H1: AI improves quality and/or efficiency without impairing the preregistered focal delayed outcome
Intermediate (comprehension, application, and structured problem-solving)	Explaining concepts, solving routine problems, applying frameworks to new cases, and procedural practice	AI as guided scaffold: worked examples and guided prompts; metacognitive scaffolds required	Moderate—AI may short-circuit the productive struggle needed for schema construction	H2: benefits contingent on metacognitive scaffolds; otherwise shallow processing prevails
Deep (analysis, evaluation, creative synthesis, and critical reflection)	Evaluating competing theories, designing novel solutions, critiquing evidence, and cross-disciplinary synthesis	AI as dialog partner: Socratic questioning and counterargument; cognitive work remains with the learner	High — outsourcing analysis, evaluation, and synthesis undermines critical-thinking development	H3 (falsifiable): product–process dissociation—higher grades but lower delayed transfer

Hypotheses H1–H3 are stated formally in Section 5.1.

Task level must be classified *a priori* in relation to the preregistered learning objective, not inferred from the observed result. Investigators should specify the objective and observable cognitive operations before randomization. A surface task delegates an operation that is not itself assessed; an intermediate task requires application of an established schema to a bounded problem; and a deep task requires learners to generate, adjudicate, justify, or integrate claims in a novel context. Informed by the ICAP framework’s distinction among modes of cognitive engagement (Chi and Wylie, 2014), two independent raters who are blind to condition and predicted direction should code each task, report weighted kappa, and resolve disagreements before data collection. The classification rule and examples should be preregistered.

### 4.1 Surface processing: AI as efficiency tool

Surface processing is defined relative to the focal learning objective rather than by an activity label alone. Information retrieval, summarization, formatting, translation, and transcription count as surface operations only when they are instrumental to, rather than constitutive of, the assessed learning goal; translation in a language-learning task, for example, may require intermediate or deep processing. When the delegated operation is extraneous to the focal objective, AI can function as an efficiency tool that frees resources for higher-order work without necessarily incurring a learning cost. The risk is comparatively low, provided that learners retain accuracy oversight and do not delegate the decision about what must be learned. Representative uses include literature searches, note organization, language polishing, reference formatting, and instrumental translation. Product-outcome studies support efficiency gains in such contexts, although the focal learning outcome must be measured separately (Mariñoso et al., 2025; Pan and Li, 2025).

### 4.2 Intermediate processing: AI as scaffold

Intermediate-level tasks involve comprehension, application, structured problem-solving, and the application of frameworks to new cases. These tasks require cognitive engagement—the learner must construct schemas linking new information to prior knowledge—but the cognitive load can be managed through well-designed scaffolding. For these tasks, we propose that AI should function as a scaffold that provides worked examples, guided prompts, and structured feedback. Critically, the effectiveness of AI at this level is contingent on the presence of metacognitive scaffolds: prompts that require learners to explain, elaborate on, or self-explain AI-generated content. Without such scaffolds, AI risks short-circuiting the productive struggle needed for schema construction—it may supply the correct answer without requiring the learner to perform the operations that produce understanding.

This proposition is directly supported by Lee and Wu's (2025) 15-week randomized controlled trial with 87 masters’ students, which demonstrated that a scaffolded ChatGPT system (SKIMIM) significantly enhanced cognitive engagement—particularly sense-making and innovation-level thinking—compared with standard ChatGPT, but only when structured prompts guided learners through knowledge integration phases (elicit initial ideas, add new concepts, distinguish viewpoints, and reflect to refine). Standard ChatGPT produced higher behavioral participation and emotional comfort but did not enhance cognitive engagement. The finding that students experienced an initial adjustment period with scaffolded AI further underscores the importance of training learners to engage productively with AI rather than defaulting to answer extraction. Dong (2026), in a study of 570 university teachers using covariance-based structural equation modeling (CB-SEM), provided convergent evidence: human–AI interaction quality reduced cognitive load, which in turn enhanced learning motivation and deep learning, with a serial mediation path explaining 53% of total variance. The dual-mediation findings suggest that AI scaffolding is most effective when it targets extraneous load reduction while preserving germane processing demands.

### 4.3 Deep processing: AI as dialog partner

Deep-level tasks involve analysis, evaluation, creative synthesis, critical reflection, and the integration of perspectives across disciplines. These tasks constitute the core of higher-order learning—the cognitive operations that define critical thinking. For these tasks, we propose that AI should function only as a dialog partner: a Socratic interlocutor that poses questions, offers counterarguments, and prompts the learner to justify, refine, and reflect on their reasoning, but does not perform the cognitive work itself. The cognitive operations of analysis, evaluation, and synthesis must remain with the learner, because it is precisely the performance of these operations that constitutes both the process and the product of higher-order learning.

The Socratic dialog approach has received preliminary empirical support. Lee et al. (2026) developed a Socratic method-enhanced ChatGPT system that replaced immediate answer provision with structured inquiry prompts and reported a shift from passive answer reception toward active knowledge construction in blended-learning discussions. Song et al. (2026), in a quasi-experimental mixed-methods study comparing a Heutagogical Deep Learning intervention with a traditional control group, reported higher deep-approach scores, lower surface-approach scores, and stronger metacognitive regulation in the intervention group. Reflective portfolios also contained instances of double-loop reflection. These studies are consistent with the possibility that carefully constrained GenAI dialog can support deeper engagement; they do not establish that a Socratic design is necessary or sufficient across domains.

The central risk at the deep-processing level—and the most dangerous manifestation of the critical thinking paradox—is that unrestricted AI use may produce a dissociation between product quality and learning. Students who use GenAI to analyze, evaluate, and synthesize may submit work of high apparent quality while having performed none of the cognitive operations that the assignment was designed to exercise. This risk is captured by our framework’s core falsifiable hypothesis (see Section 5).

### 4.4 Boundary conditions: when the framework’s predictions are moderated

Three classes of boundary conditions moderate whether AI use at each processing level follows the predicted pattern. The first is developmental stage. As reviewed in Section 2.4, primary school children tend to under-use external cognitive tools while secondary school students tend to over-use them (Sun et al., 2025), and the capacity for selective offloading develops gradually through adolescence (Armitage and Gilbert, 2025). Jing et al. (2023), in a meta-analysis of screen media and young children’s vocabulary published in Child Development, provided convergent evidence that technology effects are profoundly moderated by content quality, interactivity, and adult scaffolding. These findings imply that the framework’s risk gradient should be developmentally calibrated: younger learners may need explicit encouragement to use AI for surface tasks strategically, whereas older learners may need explicit training in recognizing when AI use becomes cognitive outsourcing. Bal and Ozturk (2025), in a systematic review, found that the cognitive effects of AI technologies on K-12 writing varied systematically by educational stage—virtual reality/augmented reality (VR/AR) tools benefited elementary students, AI assessment systems benefited middle-school students, and natural language processing (NLP) tools enhanced higher-order thinking in high-school students—reinforcing the necessity of developmental calibration.

The second boundary condition is prior knowledge. The expertise reversal effect (Kalyuga, 2007) predicts that instructional supports beneficial for novices may be redundant or even detrimental for experts. In the context of our framework, learners with low prior knowledge may benefit from greater AI scaffolding even on deep tasks, provided it is paired with self-explanation prompts and fading procedures that gradually transfer cognitive responsibility. Learners with high prior knowledge, conversely, may experience AI scaffolding as interference—the AI-generated content may disrupt their established schemas and reduce opportunities for expert-level elaboration.

The third boundary condition is metacognitive monitoring accuracy. Learners who accurately monitor their understanding may be better able to detect when AI-generated content is superficial, incorrect, or misaligned with their learning needs and adjust their use accordingly. Learners with poor calibration may be more vulnerable to habitual offloading. The proposed interaction may be self-reinforcing—repeated, weakly monitored offloading may reduce opportunities to practice calibration, which may in turn increase future offloading—but this temporal sequence has not yet been demonstrated (Choudhuri et al., 2026).

### 4.5 Metacognition as a cross-cutting mechanism

Across the recent literature, metacognition and self-regulated learning recur as a connecting theme that appears alongside every other major framework considered here—cognitive load theory, self-determination theory, cognitive offloading, critical thinking, engagement, and TPACK/AI literacy. We read this not as an artifact of keyword frequency but as a substantive reflection of the centrality of self-regulation to technology-mediated learning: metacognition functions less as a parallel tradition than as the mechanism through which the other frameworks exert their effects.

We propose that metacognitive monitoring—the capacity to accurately assess one’s own comprehension, detect gaps in understanding, and calibrate cognitive effort accordingly—operates as a cross-cutting mechanism that moderates AI’s effects at all three cognitive processing levels. At the surface level, accurate metacognitive monitoring enables learners to discriminate between tasks for which AI-assisted retrieval genuinely reduces extraneous load (adaptive offloading) and tasks for which unaided retrieval would strengthen memory traces (premature offloading). At the intermediate level, metacognitive monitoring determines whether learners spontaneously generate the self-explanations and elaborations that make AI scaffolding effective; without it, even well-designed metacognitive prompts may be processed superficially. At the deep level, metacognitive monitoring is the psychological mechanism that distinguishes episodic from habitual offloading: the learner who accurately monitors their cognitive engagement can detect when AI use has crossed the threshold from strategic delegation to unreflective dependency.

The centrality of metacognition carries a direct, falsifiable implication that extends the framework’s three primary hypotheses. We formalize this implication as Hypothesis 4 in Section 5.1.

## 5 Theoretical propositions and testable hypotheses

### 5.1 Core hypotheses derived from the differentiated framework

A central strength of the differentiated framework is that it generates falsifiable predictions—a feature that distinguishes it from general guidelines for AI integration and positions it within a testable research program. We propose three primary hypotheses, each corresponding to one level of the framework, together with the boundary-condition hypotheses that specify when the framework’s predictions are expected to hold or to be moderated.

Hypothesis 1 (Surface-Level Convergent Facilitation): When the operation delegated to AI is preregistered as extraneous to the focal learning objective, AI assistance will improve product quality and/or efficiency without producing a practically meaningful decrement in delayed performance on that focal objective relative to a matched unaided condition. H1 does not predict better recall of the information or operation that was intentionally offloaded. The hypothesis is falsified if AI assistance produces a preregistered decrement exceeding the smallest effect size of interest on the focal delayed outcome, despite equivalent task content and assessment conditions.

Hypothesis 2 (Intermediate-Level Contingent Facilitation): For tasks involving intermediate cognitive processing—comprehension, application, and structured problem-solving—the benefits of AI assistance are contingent on the presence of metacognitive scaffolds (prompts for self-explanation, elaboration, or knowledge integration). AI assistance without metacognitive scaffolds will produce higher product quality but no improvement—and potentially a decrement—in delayed transfer relative to scaffolded AI conditions. This hypothesis predicts a moderation pattern: the presence versus absence of metacognitive scaffolds determines whether AI use on intermediate tasks produces convergent facilitation or a nascent product–process dissociation. The hypothesis is falsified if unscaffolded AI use on intermediate tasks yields equivalent transfer to scaffolded AI use, which would suggest that the metacognitive demands of intermediate tasks are automatically engaged regardless of AI scaffolding.

Hypothesis 3 (Deep-Level Product–Process Dissociation): For tasks involving deep cognitive processing—analysis, evaluation, creative synthesis, and critical reflection—unrestricted AI use will produce a dissociation between product quality and learning. Specifically, AI-assisted groups will produce higher-quality assignments (as rated by expert evaluators blind to condition) but will perform significantly worse on delayed transfer tests measuring the ability to analyze, evaluate, and synthesize novel material without AI assistance. This hypothesis is the framework’s central, most falsifiable prediction. Should unrestricted AI use on deep tasks leave delayed transfer intact—or, more tellingly, enhance it—the critical thinking paradox would be challenged rather than supported. The hypothesis makes the strong prediction that the dissociation is not attributable to ceiling effects, time-on-task differences, or differential motivation, and thus requires designs that control for these confounds.

Hypothesis 4 (Metacognitive Monitoring as Cross-Level Moderator): Individual differences in metacognitive monitoring accuracy will moderate the effects predicted by H1 through H3. Specifically: (a) H1’s convergent facilitation will be weaker among learners with poor calibration, who may offload indiscriminately; (b) H2’s scaffold-contingent benefits will be weaker among learners who fail to detect comprehension gaps; and (c) H3’s product–process dissociation will be strongest among learners with the poorest monitoring accuracy. Monitoring accuracy should be measured independently of AI-use frequency through a validated performance-based calibration protocol: after each unaided transfer item, learners provide a 0–100 confidence judgment that is paired with objectively scored correctness. A preregistered primary index—such as absolute calibration error—should be supplemented by bias and discrimination (resolution) indices following Schraw (2009). The Metacognitive Awareness Inventory may be included as a secondary dispositional measure (Schraw and Dennison, 1994), but self-report awareness alone should not serve as the H4 accuracy measure. This hypothesis is consistent with intervention evidence (Lee and Wu, 2025; Song et al., 2026), but direct cross-level moderation remains to be tested.

### 5.2 The episodic–habitual offloading distinction

We propose that the transition from productive to counterproductive AI use is governed by the distinction between episodic and habitual cognitive offloading. Episodic offloading is deliberate, goal-driven, and task-specific: the learner identifies a particular cognitive operation (e.g., locating a source, formatting a reference list, and checking grammatical accuracy) and delegates it to AI while retaining metacognitive awareness of what has been offloaded and why. Habitual offloading is automatic, trust-driven, and unreflective: the learner reaches for AI as the default response to cognitive demand, without evaluating whether the specific operation being offloaded is extraneous or germane to the learning objective. We hypothesize that the transition from episodic to habitual offloading is predicted by three factors: (a) frequency of unconditioned AI use—each act of unreflective offloading reinforces the habit; (b) low metacognitive monitoring accuracy—learners who cannot detect the illusion of competence cannot correct it; and (c) environmental press—educational contexts that incentivize product quality over learning process (e.g., high-stakes grading based on assignment quality alone) create selection pressure toward habitual offloading.

### 5.3 The cognitive debt cycle

**Figure 2:**
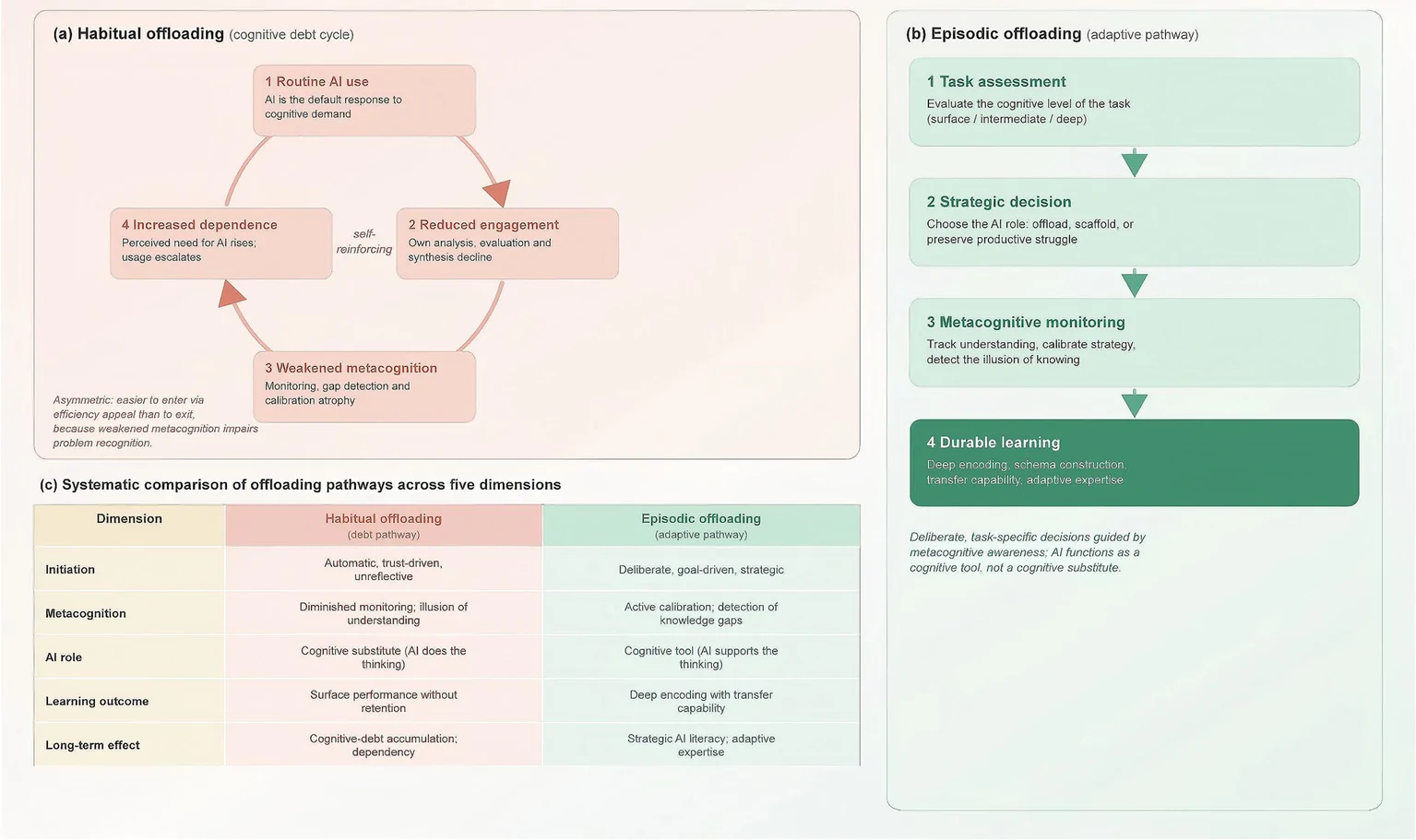
The cognitive debt cycle and the adaptive alternative. **(a)** Habitual offloading forms a self-reinforcing four-stage cycle that is easier to enter than to exit. **(b)** Episodic offloading follows a deliberate four-step pathway in which AI functions as a cognitive tool rather than a substitute. **(c)** Systematic comparison of the two pathways across five dimensions.

The cognitive debt cycle (Figure 2) is a proposed self-reinforcing mechanism through which habitual offloading could produce cumulative cognitive costs; it is not yet an established longitudinal sequence. The hypothesized stages are: (1) routine AI use, in which cognitive operations are frequently delegated; (2) reduced cognitive engagement when AI performs analysis, evaluation, or synthesis (Choudhuri et al., 2026); (3) fewer opportunities to practice metacognitive monitoring, which may weaken calibration habits through repeated disuse; and (4) increased reliance on AI as internal confidence or skill declines. Disuse-related weakening is therefore one possible mechanism, whereas dependency is a possible behavioral manifestation; neither is synonymous with cognitive debt. The proposed cycle may be asymmetric—easier to enter than to exit—and its operation should vary by the cognitive level of the offloaded task. These temporal and level-specific claims require prospective tests rather than inference from concurrent AI-use measures.

The cognitive debt cycle generates the auxiliary hypothesis that prolonged habitual offloading on deep-processing tasks will produce longitudinal declines in measures of dispositional critical thinking, intellectual humility, and need for cognition—traits that are typically conceptualized as stable individual differences, but that may, we propose, be malleable in the face of sustained cognitive offloading. This hypothesis requires longitudinal designs with baseline and follow-up measures of these dispositions, ideally tracking cohorts across the transition into AI-integrated educational environments.

### 5.4 A confirmatory test of the core prediction

Because H3 is the framework’s central and most consequential claim, we specify a concrete, pre-registrable design capable of confirming or disconfirming it. The logic of the test is a dissociation rather than a main effect: the framework predicts not merely that unrestricted AI use harms learning, but that it simultaneously improves the product and degrades the process, such that the two diverge. A design adequate to H3 must therefore measure both outcomes on the same learners and must distinguish the framework’s prescription (AI as dialog partner) from both unrestricted use and no use at all.

We propose a three-arm, between-subjects experiment (Table 2). Learners with comparable prior knowledge—verified by a pre-test and used as a covariate—are randomly assigned to complete a deep-processing assignment under one of three conditions: unrestricted AI, in which a general-purpose model may generate analysis and synthesis directly; dialog-partner AI, in which the model is constrained to Socratic questioning and counterargument but withholds the analytic work itself; and a no-AI control, in which learners use conventional resources only. Two outcomes are then measured on every learner: a product measure—assignment quality scored by at least two expert evaluators blind to condition, using a prespecified rubric with reported inter-rater reliability—and a process measure—an individually administered, unaided delayed-transfer test, given after roughly a 2-week interval, requiring analysis, evaluation, and synthesis of novel material in the same domain. We anchor the smallest dissociation of interest to roughly a 10-percentage-point transfer deficit in the unrestricted-AI arm—about d = 0.5–0.6, consistent with the d = 0.68 reported by Barcaui (2025)—which, at *α* = 0.05 and 80% power, implies approximately 45–64 participants per arm; we recommend recruiting approximately 80 per arm to accommodate attrition and covariate adjustment.

**Table 2:** Proposed three-arm experimental design for a confirmatory test of H3 (deep-level product–process dissociation).

Design element	Specifications
Participants	Undergraduates with comparable prior knowledge, verified by a pre-test (covariate). *A priori* power for an independent-groups contrast (α = 0.05, power = 0.80) requires ≈ 64 per group to detect d = 0.5 and ≈ 45 to detect d = 0.6; recruiting ≈ 80 per group (≈ 240 total) buffers attrition and the covariate-adjusted interaction test.
Task	An *a priori* deep-processing assignment, classified under the preregistered Section 4 decision rule by two independent raters blind to condition and predicted direction, requiring analysis, evaluation, and synthesis of novel material.
Arm 1—Unrestricted AI	Learners may use a general-purpose LLM without constraint, including to generate analysis, evaluation, and synthesis directly.
Arm 2—Dialog-partner AI	Learners may use an LLM constrained to Socratic functions (questions, counterarguments, and requests for justification) that does not supply analysis, evaluation, or synthesis.
Arm 3—No-AI control	Learners complete the task with conventional resources (library databases and readings) but no generative AI.
Product measure	Assignment quality rated by ≥2 expert evaluators blind to condition, using a prespecified analytic rubric; inter-rater reliability reported (target ICC ≥ 0.80).
Process/learning measure	Individually administered, unaided delayed transfer test (~ 2-week interval) requiring analysis, evaluation, and synthesis of novel material in the same domain; scored blind to condition.
Controls for confounds	Fixed by design: developmental or educational stage, domain and task materials, GenAI model/version, system instructions, access duration, task framing, and assessment rubric. Measured *a priori* or during the task: prior knowledge, baseline monitoring calibration, prior AI experience, time-on-task, state motivation, and cognitive load. Only preregistered covariates and moderators enter confirmatory models; interaction logs verify manipulation fidelity.
Analysis	Mixed-design model testing the Condition × Measure-type interaction; planned contrasts: Arm 1 > Arms 2–3 on product but Arm 1 < Arms 2–3 on delayed transfer; equivalence testing to interpret null transfer differences.

Arm 2 operationalizes the framework’s prescription for deep-processing tasks; the predicted ordering across all three arms, not a single pairwise difference, is what distinguishes H3 from rival accounts.

H3 predicts a crossover: the unrestricted AI arm should produce the highest-rated assignments yet the lowest delayed-transfer scores, while the dialog partner arm should sacrifice little or no product quality while preserving—or improving—transfer relative to unrestricted use. The design addresses rival explanations directly: prior knowledge is assessed before randomization; time-on-task is logged; transfer difficulty is piloted to avoid ceiling effects; and motivation and cognitive load are measured rather than assumed equal. Substituting surface- and intermediate-level assignments yields parallel tests of H1 and H2 within the same measurement structure. Confirmatory tests should use registered reports so that an absent or reversed dissociation—which would challenge the framework—is as publishable as a positive result.

To prevent boundary conditions from becoming *post hoc* explanations, the primary H3 test fixes developmental or educational stage, domain and task materials, GenAI model and version, system instructions, access duration, task framing, and assessment rubric across conditions. Prior knowledge, baseline monitoring calibration, prior AI experience, time-on-task, state motivation, and cognitive load are measured using instruments specified before data collection. The unadjusted Condition × Measure-type interaction and planned arm ordering constitute the primary confirmatory test; covariate-adjusted and moderated models are secondary and preregistered. A failed or reversed primary interaction cannot be reclassified after inspection of the boundary measures. Boundary conditions should then be tested prospectively through planned stratification or replication across populations.

## 6 Methodological implications for future research

The critical-thinking paradox carries direct methodological implications. The 2024–2025 evidence base illustrates why product-only outcome measures can yield incomplete conclusions about GenAI’s educational effects. Studies that measure only assignment grades, satisfaction, or perceived learning cannot determine whether product quality and durable, unaided performance converge or dissociate.

We propose five methodological recommendations. First, future studies should incorporate multimodal, process-sensitive measures alongside traditional product outcomes. Adnans et al. (2025), in a study of 665 students examining ChatGPT-enhanced collaborative learning through a Bloom’s Taxonomy lens, combined Partial Least Squares Structural Equation Modeling, Necessary Condition Analysis, and fuzzy-set Qualitative Comparative Analysis. This approach identified understanding, rather than mere recall, as a necessary condition for critical thinking development. GenAI learning research should routinely incorporate at least two classes of dependent variables: product measures (e.g., assignment quality and completion time) and process or learning measures (e.g., cognitive engagement, monitoring calibration, and unaided delayed transfer). Jarvela and Hadwin (2024) outlined a multimodal framework for self-regulated learning research that integrates physiological data, interaction traces, and situational self-reports and can be adapted to GenAI-mediated learning.

Operational telemetry can make the episodic–habitual transition observable in real time. With informed consent and privacy-preserving storage, chat logs should yield preregistered indicators, such as prompt timestamps, session duration, the proportion of direct-answer versus explanation or critique prompts, prompt revisions, follow-up questions, copy or paste events, and latency before requesting AI assistance. Brief Experience Sampling Methodology prompts delivered after sampled interactions can record the learner’s immediate goal, perceived effort, whether AI use was deliberate or automatic, confidence, and ability to explain the output without assistance. A transition should be inferred only when repeated sessions across tasks show increasing default or direct-answer prompting together with declining deliberateness and poorer unaided performance or calibration; chat-log frequency alone measures offloading, not cognitive debt. Multilevel time-series or latent-transition models can link within-person telemetry to subsequent unaided outcomes, using a preregistered coding ontology and human reliability checks for prompt classification.

Second, we recommend that the field adopt registered report formats for confirmatory tests of the dissociation hypothesis. Registered reports, in which study designs and analysis plans are peer-reviewed before data collection, minimize publication bias and ensure that null results—including the absence of dissociation, which would challenge the framework—are published alongside positive findings. Third, longitudinal designs are essential to determine whether the cognitive debt cycle represents a transient adjustment period or a durable recalibration of cognitive habits. Xia (2025), employing cross-lagged panel modeling with latent profile analysis, demonstrated that self-regulated learning and deep approaches to learning interact dynamically over time in GenAI-mediated environments, with early reflection predicting later forethought. Such designs, extended across longer time intervals and tracking dispositional cognitive measures, can adjudicate between competing accounts of the cognitive debt hypothesis. Fourth, experimental designs that manipulate the presence versus absence of metacognitive scaffolds at each processing level can provide direct tests of Hypothesis 2. In contrast, designs that manipulate AI availability on deep-processing tasks with delayed transfer as the primary outcome can test the framework’s central Hypothesis 3.

Fifth, the verified corpus contains a substantial body of controlled intervention research examining cognitive load and metacognitive supports in AI-mediated educational settings, providing a developing experimental toolkit for framework testing. Because the scoping review identifies method terminology by keyword coding rather than full-text design adjudication, we do not treat the keyword count as a confirmed number of randomized controlled trials. We specifically recommend a 2 (AI availability: unrestricted vs. none) x 2 (metacognitive scaffold: present vs. absent) x 2 (cognitive level: intermediate vs. deep) factorial design with delayed transfer as the primary outcome, which would simultaneously test H2, H3, and H4 within a single experimental paradigm. Barcaui’s (2025) 45-day delayed retention RCT (*n* = 120, d = 0.68) provides a directly adaptable methodological template for the deep-processing condition.

## 7 Implications for teacher education and educational policy

The mediation gap identified in Section 2.3—the finding that AI knowledge influences AI integration only through the mediation of pedagogical beliefs and attitudes—carries direct implications for teacher preparation. Current professional development programs, including those evaluated by Han (2025) and Kazmaci et al. (2025), prioritize the transmission of technical knowledge over the cultivation of adaptive beliefs and critical AI literacy. Our framework suggests that effective teacher preparation must address three components: AI-technological knowledge (how GenAI tools function), AI-pedagogical knowledge (how to classify learning tasks by cognitive level and match AI strategies accordingly), and AI-critical knowledge (when not to use AI, grounded in understanding of productive struggle and the episodic–habitual distinction). This tripartite structure extends Shulman's (1986) pedagogical content knowledge construct into the GenAI era and aligns with emerging Intelligent-TPACK frameworks (Al-Abdullatif, 2024; Wang et al., 2024; Chen et al., 2025) that specify AI technological knowledge, AI technological pedagogical knowledge, and AI ethical knowledge as distinct competencies.

Concretely, the differentiated framework offers a curricular heuristic: teacher candidates can be trained to classify learning tasks by cognitive level using the surface–intermediate–deep taxonomy presented in Table 1, and to select AI-integration strategies corresponding to each level. Training should explicitly include practice in designing metacognitive scaffolds for intermediate tasks—a skill that Lee and Wu (2025) demonstrated can be operationalized through the Scaffolded Knowledge Integration framework—and in deploying Socratic questioning strategies for deep tasks. Critically, training must also address the episodic–habitual distinction: teachers should develop the ability to recognize when students are transitioning from strategic AI use (episodic offloading) to unreflective dependency (habitual offloading), and to intervene through metacognitive prompts, task redesign, or scaffold fading.

For policymakers, the evidence reviewed here counsels caution about uncritical mandates for AI integration in curricula. If AI adoption widens rather than narrows the gap between surface and deep learning, educational systems may produce students who perform well on routine assessments yet lack the critical thinking capacities that higher education, professional practice, and democratic citizenship demand. The developmental evidence reviewed in Sections 2.4 and 4.4 indicates that blanket AI integration policies applied uniformly across grade levels may have developmentally inappropriate effects. Policy should incentivize the development of AI literacy frameworks that distinguish among cognitive processing levels and developmental stages, rather than treating AI integration as a uniform good. At the institutional level, assessment policies that reward product quality without measuring the learning process may inadvertently select for the habitual offloading pathway. Assessment reform—including the incorporation of process portfolios, oral defenses, and in-class, unaided performance tasks alongside traditional assignments—may be necessary to align institutional incentives with the goal of preserving cognitive engagement in AI-integrated education.

## 8 Conclusion

The critical-thinking paradox does not imply that GenAI should be excluded from education. GenAI’s educational potential is more likely to be realized when deployment is guided by theoretically grounded, empirically testable distinctions between the operation delegated to AI and the focal learning objective. The first wave of evidence, concentrated in 2024–2025, has surfaced a candidate paradox: under some conditions, GenAI may improve academic products while reducing the cognitive activity that supports deep, durable learning. Under other conditions, product and learning gains may converge. The next wave of research must distinguish these possibilities.

To the best of our knowledge, the differentiated framework proposed here is among the first to operationalize the AI-learning paradox through cognitive-processing levels in a way that yields testable predictions about when GenAI serves versus undermines learning. Its three level-specific hypotheses—surface convergent facilitation, intermediate contingent facilitation, and deep product–process dissociation—together with H4’s cross-level metacognitive moderator provide a program for experimental, quasi-experimental, telemetry-based, and longitudinal research. The episodic–habitual distinction and three-level mapping are the principal conceptual extensions advanced here. Cognitive debt, adopted from the emerging literature, is treated as a downstream longitudinal outcome that must be measured independently of offloading behavior through changes in unaided performance and calibration.

Educational psychologists are uniquely positioned to lead this study. The discipline possesses the theoretical resources—from levels of processing and desirable difficulties to cognitive load theory and self-determination theory—to develop the conceptual frameworks, measurement tools, and pedagogical guidelines that allow AI integration to serve learning rather than merely simulate it. The critical thinking paradox is not an argument against GenAI in education; it is an argument for the centrality of educational psychology in ensuring that GenAI’s integration into educational settings is guided by evidence, grounded in theory, and oriented toward the preservation and cultivation of the cognitive capacities that define educated thought. The first wave of evidence is in; the task now is to ensure that the second wave asks better questions, deploys better methods, and generates better answers.

## Data Availability

The original contributions presented in the study are included in the article/Supplementary material, further inquiries can be directed to the corresponding author.
