# Supplementary material for: The critical-thinking paradox in generative AI-integrated learning: distinguishing efficiency from cognitive depth—a differentiated framework and testable propositions
Source: Front Psychol. 2026 Aug 14;17:1906070. doi: 10.3389/fpsyg.2026.1906070 (PMC13521781; doi:10.3389/fpsyg.2026.1906070)
Supplement: Supplementary file 1 [file Supplementary_file_1.PDF]

Supplementary Material

Bibliometric Scoping Review: Reproducible Methods and VALOR Compliance

This Supplement documents the complete, reproducible methodology for the bibliometric scoping review referenced in Section 2.5 of the main article.

Reporting standard. This Supplement adapts the VALOR framework—Verification, Alignment, Logging, Overview, and Reproducibility (Hoang, 2025)—to a single-database, multi-query scoping review. Table S1 maps each VALOR dimension to the relevant section and records the completed consistency checks.

Table S1. VALOR compliance summary

Each row states the VALOR dimension, how it is satisfied in this study, the supporting location, and the result of the verification check.

VALOR dimension	How addressed in this study	Location	Verification check / status
Verification (data consistency)	Record counts were independently reconciled across every retrieval and screening stage; the final year distribution and 170-title SSCI whitelist were rechecked against the auditable workbook.	§2, §7; Tables S2, S6	PASS. Year counts sum exactly to 969. The whitelist contains 170 canonical titles and three verified aliases. All stage deltas are exact.
Alignment (time synchronization)	All 12 finalized queries were re-executed on one date against Scopus, with query-specific year windows stated explicitly.	§1.1–1.2; Table S2	PASS. Single database and final verification search date: 25 July 2026. The 2026 count is explicitly identified as year-to-date.
Logging (documentation)	Final query strings, raw per-query counts, exported fields, deduplication keys, eligibility criteria, exclusion codes, and coding schemes are recorded.	§1–5; Tables S2–S5	PASS. Twelve finalized queries and all deterministic coding patterns are logged.
Overview (result validation)	Outputs are interpreted within the limits of keyword coding, SSCI restriction, small base-year counts, and a single-database design.	§5–6, §9	PASS with caveats. Deep/surface disambiguation and partial-year interpretation are explicitly documented.
Reproducibility (re-execution)	A reader with authorized Scopus access can re-execute the finalized queries; coding and screening rules are fully specified.	§1–5, §7–8	PASS. Raw CSVs are retained but not publicly redistributed; the verified dataset, screening workbook, whitelist, and coding matrix are available on request.

Note. *"PASS" indicates the dimension is documented and its internal consistency check succeeded; "PASS with caveats" indicates documented limitations that bound interpretation (detailed in §9). This scoping review has a contextualizing function in the main article and is not a standalone systematic review or meta-analysis.*

1. Search strategy

1.1 Database and date

Database: Scopus (Elsevier). Final verification search date: 25 July 2026. Access: Scopus was accessed through the corresponding author's personal account under a self-funded subscription. Export format: CSV, all available fields.

1.2 Search queries

As part of a revision-stage verification and reproducibility check, the 12 finalized queries were re-executed independently on 25 July 2026. Each final query is reproduced verbatim below with its publication-year window and raw export count. Scopus Advanced Search syntax is retained exactly; W/n denotes proximity, PUBYEAR AFT/BEF sets exclusive year bounds, and * is the wildcard.

Query 1 — GenAI + Critical Thinking + Education (2021–2026) (raw n = 2,620)

```
TITLE-ABS-KEY(("generative artificial intelligence" OR "generative AI" OR ChatGPT OR "large language model*" OR LLM*) AND ("critical thinking" OR "cognitive engagement" OR "cognitive depth" OR "higher-order thinking" OR "higher order thinking" OR "cognitive disengagement" OR "cognitive erosion") AND (educat* OR student* OR learner* OR teach* OR classroom* OR school* OR universit* OR "higher education" OR "learning outcome*" OR "learning process*" OR "academic achievement")) AND PUBYEAR AFT 2020 AND PUBYEAR BEF 2027
```

Query 2 — Cognitive Offloading + AI + Learning (2016–2026) (raw n = 232)

```
TITLE-ABS-KEY ( ( "cognitive offloading" OR "cognitive outsourcing" OR "cognitive dependency" OR "cognitive externalization" OR "cognitive externalisation" OR "cognitive crutch" OR "metacognitive laziness" ) AND ( "artificial intelligence" OR "AI" OR "digital technolog*" OR "generative AI" OR ChatGPT OR "large language model*" ) AND ( learning OR education OR memory OR retention OR metacognition OR "self-regulated learning" ) ) AND PUBYEAR AFT 2015 AND PUBYEAR BEF 2027
```

Query 3 — Deep/Surface Approaches, GenAI, and Individual Differences (2016–2026) (raw n = 18)

```
TITLE-ABS-KEY (((deep W/4 (learn* OR approach* OR process* OR strateg*)) AND (surface W/4 (learn* OR approach* OR process* OR strateg*)) OR "study process questionnaire" OR "revised two-factor study process questionnaire" OR "R-SPQ-2F" OR "approaches and study skills inventory for students") AND ("generative artificial intelligence" OR "generative AI" OR ChatGPT OR "large language model*" OR LLM* OR "GPT-3" OR "GPT-4") AND (education* OR student* OR learner* OR universit* OR "higher education" OR classroom* OR school* OR "individual difference*" OR "student agency" OR metacognit* OR self-regulat* OR motivation* OR "prior knowledge")) AND PUBYEAR AFT 2015 AND PUBYEAR BEF 2027
```

Query 4 — Teacher AI Literacy + AI-TPACK + Teacher Education (2019–2026) (raw n = 1,254)

```
TITLE-ABS-KEY ( ( "AI literacy" OR "artificial intelligence literacy" OR "AI-TPACK" OR "Intelligent TPACK" OR "AI pedagogical content knowledge" OR "AI competency" OR "AI competence" ) AND ( teacher* OR "pre-service" OR "preservice" OR "in-service" OR "teacher education" OR "teacher training" OR "professional development" ) AND ( "artificial intelligence" OR "generative AI" OR ChatGPT OR "AI integration" ) ) AND PUBYEAR AFT 2018 AND PUBYEAR BEF 2027
```

Query 5 — Cognitive Load Theory + AI + Education (2016–2026) (raw n = 1,160)

```
TITLE-ABS-KEY ( ( "cognitive load theory" OR "cognitive load" OR "extraneous load" OR "germane load" OR "intrinsic load" OR "load reduction instruction" ) AND ( "artificial intelligence" OR "generative AI" OR ChatGPT OR "AI-assisted" OR "AI-supported" OR "AI-integrated" OR "LLM*" ) AND ( education OR learning OR instruction* OR student* OR classroom* ) ) AND PUBYEAR AFT 2015 AND PUBYEAR BEF 2027
```

Query 6 — GenAI Education Paradox / Double-Edged Effects / Cognitive Debt (2020–2026) (raw n = 1,067)

```
TITLE-ABS-KEY (("generative artificial intelligence" OR "generative AI" OR ChatGPT OR "large language model*" OR LLM* OR "artificial intelligence in education" OR "AI in education") AND (paradox* OR "double-edged" OR "double edged" OR "double-edged sword" OR "dual effect*" OR "cognitive debt" OR "cognitive paradox" OR "productivity paradox" OR "trade-off*" OR tradeoff* OR (benefit* W/5 risk*) OR (enhanc* W/8 erod*)) AND (education* OR learn* OR student* OR learner* OR teach* OR classroom* OR academic* OR "critical thinking" OR "cognitive engagement" OR metacognit*)) AND PUBYEAR AFT 2019 AND PUBYEAR BEF 2027
```

Query 7 — Metacognition + AI + Self-Regulated Learning (2016–2026) (raw n = 1,248)

```
TITLE-ABS-KEY ((metacognit* OR "metacognitive regulation" OR "metacognitive calibration" OR "self-regulated learning" OR "self regulated learning") AND ("generative artificial intelligence" OR "generative AI" OR ChatGPT OR "large language model*" OR LLM* OR "artificial intelligence in education" OR "AI in education" OR "AI-assisted learning" OR "AI-supported learning" OR "intelligent tutoring system*") AND (education* OR learn* OR student* OR learner* OR teach* OR universit* OR classroom* OR academic*)) AND PUBYEAR AFT 2015 AND PUBYEAR BEF 2027
```

Query 8 — Self-Determination Theory + AI + Education (2016–2026) (raw n = 452)

```
TITLE-ABS-KEY (( "self-determination theory" OR "self determination theory" OR "basic psychological need*" OR "psychological need satisfaction" OR "psychological need frustration" OR "autonomous motivation" OR "controlled motivation" OR ((autonomy OR "autonomy support*") AND competence AND relatedness) ) AND ("artificial intelligence" OR "generative artificial intelligence" OR "generative AI" OR ChatGPT OR "large language model*" OR LLM* OR "AI chatbot*" OR "AI tutor*" OR "intelligent tutoring system*") AND (education* OR learn* OR student* OR learner* OR teach* OR classroom* OR universit* OR "academic engagement" OR "student well-being")) AND PUBYEAR AFT 2015 AND PUBYEAR BEF 2027
```

Query 9 — Five Target Journals Precision Search (2016–2026) (raw n = 96)

```
( SRCTITLE ( "Child Development" ) OR SRCTITLE ( "School Psychology" ) OR SRCTITLE ( "School Psychology Review" ) OR SRCTITLE ( "Journal of Counseling Psychology" ) OR SRCTITLE ( "Learning and Individual Differences" ) ) AND TITLE-ABS-KEY ( ( "artificial intelligence" OR "AI" OR "generative AI" OR ChatGPT OR "large language model*" OR "machine learning" OR "digital technolog*" OR "cognitive offloading" ) AND ( learning OR education OR cognition OR cognitive OR student* OR child* OR adolescent* OR development OR metacognition ) ) AND PUBYEAR AFT 2015 AND PUBYEAR BEF 2027
```

Query 10 — Children/Adolescents + Digital Technology + Cognitive Development (2016–2026) (raw n = 689)

```
TITLE-ABS-KEY ( ( child* OR adolescent* OR "young child*" OR "early childhood" OR "primary school" OR
```

```
"secondary school" OR "middle childhood" ) AND ( "digital technolog*" OR "screen media" OR touchscreen OR "AI" OR "artificial intelligence" OR "educational technolog*" ) AND ( "cognitive development" OR "executive function*" OR "attention control" OR "self-regulation" OR metacognition OR "cognitive offloading" ) ) AND PUBYEAR AFT 2015 AND PUBYEAR BEF 2027
```

Query 11 — Cognitive Offloading + Child Development (2016–2026) (raw n = 77)

```
TITLE-ABS-KEY ( ( "cognitive offloading" OR "offloading" OR "external memory" OR "external representation*" ) AND ( child* OR "young child*" OR "primary school" OR "elementary school" OR "middle school" OR "secondary school" OR adolescent* ) AND ( metacognition OR "metacognitive monitoring" OR "memory" OR "strategy use" OR development ) ) AND PUBYEAR AFT 2015 AND PUBYEAR BEF 2027
```

Query 12 — GenAI Hallucination/Over-trust + Learning (2021–2026) (raw n = 1,333)

```
TITLE-ABS-KEY ( ( "AI hallucination*" OR "LLM hallucination*" OR "model hallucination*" OR "hallucinated information" OR "over-trust" OR overtrust OR "overreliance" OR "over-reliance" OR "uncritical reliance" OR "automation bias" OR "algorithm aversion" OR "algorithmic reliance" OR "trust calibration" OR "calibrated trust" OR "illusion of competence" OR "illusion of understanding" ) AND ( "generative artificial intelligence" OR "generative AI" OR ChatGPT OR "large language model*" OR LLM* OR "AI-generated content" OR "AI chatbot*" OR "AI-assisted learning" ) AND ( educat* OR student* OR learner* OR teach* OR classroom* OR school* OR universit* OR "higher education" OR "student learning" OR "learning outcome*" OR "information literacy" OR "critical thinking" OR metacognit* ) ) AND PUBYEAR AFT 2020 AND PUBYEAR BEF 2027
```

1.3 Exported fields

Per Scopus CSV export: authors, title, year, source title, document type, language, abstract, author keywords, index keywords, DOI, EID, cited-by count, publication stage, link, and other available citation and bibliographic fields.

2. Deduplication and filtering pipeline

Table S2 records the seven-stage cascade from raw retrieval to the verified final corpus. The "Removed" column reports each exact stage-to-stage delta.

Table S2. Record counts at each stage of the screening pipeline

Stage	Criterion	Retained	Removed	Deduplication / exclusion detail
—	Raw retrieval from 12 finalized queries	10,246	—	Sum of the 12 raw exports: 2,620 + 232 + 18 + 1,254 + 1,160 + 1,067 + 1,248 + 452 + 96 + 689 + 77 + 1,333.
1	Within-query duplicate removal	10,201	45	DOI-first deduplication; normalized-title fallback was used only when DOI evidence did not conflict.
2	Cross-query merge and deduplication	8,869	1,332	Overlapping records retrieved by multiple queries were merged while preserving all contributing Query IDs.
3	Document type = Article OR Review	5,269	3,600	Conference papers/reviews, book chapters/books, editorials, notes, letters, errata, short surveys, data papers, retracted items, and other types were excluded.
4	English-language original document	5,126	143	Records whose original-document language was not English were excluded.
5	SSCI 2024 whitelist match	1,080	4,046	Exact match to 170 canonical source titles or three verified aliases; unlisted, ESCI-only, and SCIE-only non-SSCI sources were excluded.
6	Title/abstract relevance screening	969	111	Manual exclusions: E06 wrong domain/context (37), E07 no qualifying exposure (52), E08 no target construct (19), E10 clinical AI only (2), and E11 business/workplace AI only (1).

Note. All values are exact. The deduplication stages reconcile as 10,246 − 45 − 1,332 = 8,869; the eligibility and relevance stages reconcile as 8,869 − 3,600 − 143 − 4,046 − 111 = 969. The final corpus (n = 969) is the basis for the contextual bibliometric statements in Section 2.5 of the main article.

3. Coding scheme for theoretical frameworks

Each of the 969 included publications was coded for the presence of each framework by keyword matching on a concatenated, lowercased field comprising Author Keywords, Index Keywords, Title, and Abstract. A publication

scored 1 for a framework if any listed pattern matched and 0 otherwise. Coding is deterministic and re-executable from the patterns below.

Table S3. Keyword patterns for theoretical-framework coding

Framework	Keyword matching patterns (case-insensitive; * = wildcard)
Cognitive Offloading	"cognitive offloading" · "cognitive off-loading" · "cognitive outsourcing" · "cognitive externali*" · "epistemic offloading" · "cognitive dependency" · "offload*"
Metacognition / SRL	"metacognit*" · "self-regulated learning" · "self-regulation" · "SRL" · "metacognitive monitoring" · "metacognitive awareness" · "metacognitive strateg*"
Cognitive Load Theory	"cognitive load" · "extraneous load" · "germane load" · "intrinsic load" · "CLT" · "load reduction instruction"
Self-Determination Theory	"self-determination theory" · "SDT" · "basic psychological need*" · "autonomy support*" · "intrinsic motivation" · "self-determined"
Engagement Theory	"student engagement" · "cognitive engagement" · "behavioral engagement" · "emotional engagement" · "agentic engagement" · "academic engagement"
TPACK / AI-TPACK	"TPACK" · "technological pedagogical content knowledge" · "AI-TPACK" · "Intelligent TPACK" · "AI pedagogical content knowledge"
Critical Thinking	"critical thinking" · "higher-order thinking" · "analytical thinking" · "argumentation"
Deep/Surface Approaches	"deep approach*" · "surface approach*" · "approaches to learning" · "SPQ" · "R-SPQ" · "study process questionnaire"
Desirable Difficulties	"desirable difficult*" · "Bjork" · "retrieval practice" · "spacing effect" · "testing effect" · "generation effect"
Levels of Processing	"levels of processing" · "depth of processing" · "Craik and Lockhart"

Note on deep/surface disambiguation. For "Deep/Surface Approaches," machine-learning uses of "deep learning" were excluded by requiring an education-specific learning-approach term. This conservative rule yielded n = 3 in the final corpus and should be interpreted as a lower-bound indicator rather than the true prevalence of depth-of-processing research.

4. Coding scheme for statistical methods

Statistical methods were coded by the same deterministic keyword-matching procedure on the same concatenated fields in the final corpus (n = 969).

Table S4. Keyword patterns for statistical-method coding

Method	Keyword matching patterns (case-insensitive; * = wildcard)
SEM (general)	"structural equation model*" · "SEM" · "path analysis" · "confirmatory factor analysis" · "CB-SEM"
PLS-SEM	"PLS-SEM" · "partial least squares" · "PLS path model*" · "SmartPLS"
Longitudinal / CLPM	"longitudinal" · "cross-lagged" · "CLPM" · "panel model*" · "repeated measure*" · "latent growth"
Mixed Methods	"mixed method*" · "mixed-method*" · "convergent design" · "explanatory sequential" · "triangulation"
Meta-Analysis	"meta-analysis" · "meta analysis" · "systematic review" · "meta-analytic" · "pooled effect" · "forest plot"

Method	Keyword matching patterns (case-insensitive; * = wildcard)
RCT	"randomized controlled" · "RCT" · "randomly assigned" · "treatment group" · "control group"
Latent Profile / Class	"latent profile analysis" · "latent class analysis" · "LPA" · "LCA" · "person-centered" · "growth mixture"
fsQCA / NCA	"fsQCA" · "qualitative comparative analysis" · "fuzzy set" · "necessary condition analysis" · "NCA"
Bayesian	"Bayesian" · "Bayes factor" · "MCMC" · "credible interval" · "posterior distribution"
Machine Learning	"machine learning" · "random forest" · "neural network" · "SVM" · "classification algorithm"
Rasch / IRT	"Rasch model" · "item response theory" · "IRT" · "differential item functioning"

Note. "SEM (general)" and "PLS-SEM" are coded separately, and method codes are not mutually exclusive. These keyword indicators identify candidate design terminology; they are not substitutes for full-text design adjudication. Accordingly, the main article does not report a keyword-derived count as a confirmed number of randomized controlled trials.

5. Co-occurrence and growth-rate computation

Co-occurrence. Theory–theory and method–theory co-occurrence were computed per publication in the final corpus (n = 969). For each unordered pair whose two binary codes equaled 1, the corresponding cell was incremented by 1. Co-occurrence therefore denotes joint keyword presence within a publication, not substantive theoretical integration.

Table S5. Growth-rate (CAGR) computation rules

Element	Definition
**Formula**	$\mathbf{CAGR = [\ (Count_{2026} / Count_{base})\^{}(1/n) - 1\ ] \times 100\%}$
Base year	2021 where the 2021 count > 0; otherwise 2022 (the first year with a non-zero count).
n	Number of years from the base year to 2026 (e.g., base 2022 → n = 4).
"N/A (from ~0)"	Reported when the base-year count is 0, so a multiplicative growth rate is undefined; the trend is described qualitatively instead.
2026 caveat	2026 is a partial, year-to-date count at the 25 July 2026 final verification search date. Growth estimates ending in 2026 are interpreted as directional rather than as full-year rates.

6. Year distribution of the final corpus (consistency check)

Table S6 reports the publication-year distribution of the verified final corpus. The year counts sum exactly to 969, providing the primary internal-consistency check for VALOR Verification (Table S1).

Table S6. Publication-year distribution (n = 969)

Year	2016	2017	2018	2019	2020	2021	2022	2023	2024	2025	2026 (YTD)
Records	1	7	2	5	6	4	13	32	69	350	480

Check: 1 + 7 + 2 + 5 + 6 + 4 + 13 + 32 + 69 + 350 + 480 = 969. The 2025–2026 entries together account for 830 records (85.7% of the corpus). The 2026 value is year-to-date at the final verification search date.

7. SSCI journal whitelist (170 journals)

Stage 5 retained only records whose source title matched one of 170 canonical Scopus source titles in the

prespecified Web of Science Social Sciences Citation Index (SSCI) 2024 whitelist or one of three verified title aliases. The whitelist covers Education & Educational Research; Psychology, Educational; Psychology, Developmental; Psychology, Experimental; Psychology, Multidisciplinary; and selected adjacent SSCI journals relevant to educational technology.

Table S7. Representative journals from the whitelist (15 of 170 shown)

Journal	Journal
Computers and Education	British Journal of Educational Technology
Journal of Computer Assisted Learning	Interactive Learning Environments
Education and Information Technologies	Educational Technology Research and Development
Journal of Educational Computing Research	Technology, Pedagogy and Education
Journal of Research on Technology in Education	Computers in Human Behavior
Internet and Higher Education	Australasian Journal of Educational Technology
British Journal of Educational Psychology	Learning and Individual Differences
Child Development	

Exclusion note. *ESCI (Emerging Sources Citation Index) journals were excluded because they lack a Journal Impact Factor in the SSCI edition, even where they publish education-technology research of comparable quality (e.g., Education Sciences, Frontiers in Education, Cogent Education). This is a deliberate, documented boundary, not an omission (see §9c).*

8. Data availability

The verified final dataset contains 969 publications and includes authors, title, year, source title, document type, language, abstract, DOI, EID, query provenance, and author/index keywords as available in Scopus. DOI and keyword fields are reported where indexed. The dataset, screening workbook, SSCI whitelist, and coding matrix are available from the corresponding author on reasonable request. The 12 raw Scopus CSV exports are retained by the authors but are not publicly redistributable under Scopus licensing terms; the finalized queries in Section 1.2 permit re-execution through an authorized Scopus account.

9. Limitations

This scoping review has a contextualizing function in the accompanying article (Section 2.5); it is not a standalone systematic review or meta-analysis. Specific limitations:

(a) Single database. Retrieval used Scopus only; Web of Science, ERIC, and PsycINFO were not searched, so coverage is bounded by Scopus indexing.

(b) Keyword-based coding. Coding matched keywords rather than full text, which may underestimate framework presence in publications that use a construct without listing it as a keyword. In addition, two coded frameworks — Desirable Difficulties and Levels of Processing — were not themselves retrieval targets in any of the twelve queries; their corpus frequencies are therefore lower bounds on prevalence and are interpreted qualitatively only (cf. main article, Sections 3.1–3.2).

(c) ESCI exclusion. The SSCI whitelist excludes ESCI journals that publish AI-education research of comparable quality; the corpus therefore under-represents very recent or emerging venues.

(d) Unstable small-base growth. CAGR estimates for frameworks with very small base-year counts are numerically unstable and are interpreted as trend direction rather than precise magnitude.

(e) Co-occurrence ≠ integration. Co-occurrence reflects joint research attention in keyword space, not necessarily substantive theoretical integration within the cited publications.

(f) Revision-stage query verification. During revision, the query wording was refined to improve construct precision, disambiguate deep/surface learning from neural-network terminology, and apply an explicit upper publication-year bound. All 12 finalized queries were then re-executed on 25 July 2026. The reported 10,246-record raw pool and 969-publication final corpus derive exclusively from these finalized queries; no results from earlier exploratory versions were retained.

Reference

Hoang, A.-D. (2025). Evaluating bibliometrics reviews: A practical guide for peer review and critical reading. *Evaluation Review* **49**(6), 1074–1102. doi: 10.1177/0193841X251336839
